# Supplementary material for: Cullin 3 Exon 9 Deletion in Familial Hyperkalemic Hypertension Impairs Cullin3-Ring-E3 Ligase (CRL3) Dynamic Regulation and Cycling
Source: Int J Mol Sci. 2022 May 5;23(9):5151. doi: 10.3390/ijms23095151 (PMC9105235; doi:10.3390/ijms23095151)
Supplement: Supplementary file 1 [file ijms-23-05151-s001.zip › SUPPLEMENTAL FIGURES.pdf]

International Journal of
Molecular Sciences

MDPI

SUPPLEMENTAL FIGURES

Cullin 3 Exon 9 Deletion in Familial Hyperkalemic Hypertension Impairs Cullin3-Ring-E3 Ligase (CRL3) Dynamic Regulation and Cycling

Ilektra Kouranti [1], Waed Abdel-Khalek [1], Stephani Mazurkiewicz [1], Irmine Loisel-Ferreira [1], Alexis M. Gautreau [2,3], Lionel Pintard [4], Xavier Jeunemaitre [1] and Eric Clauser [1,*]

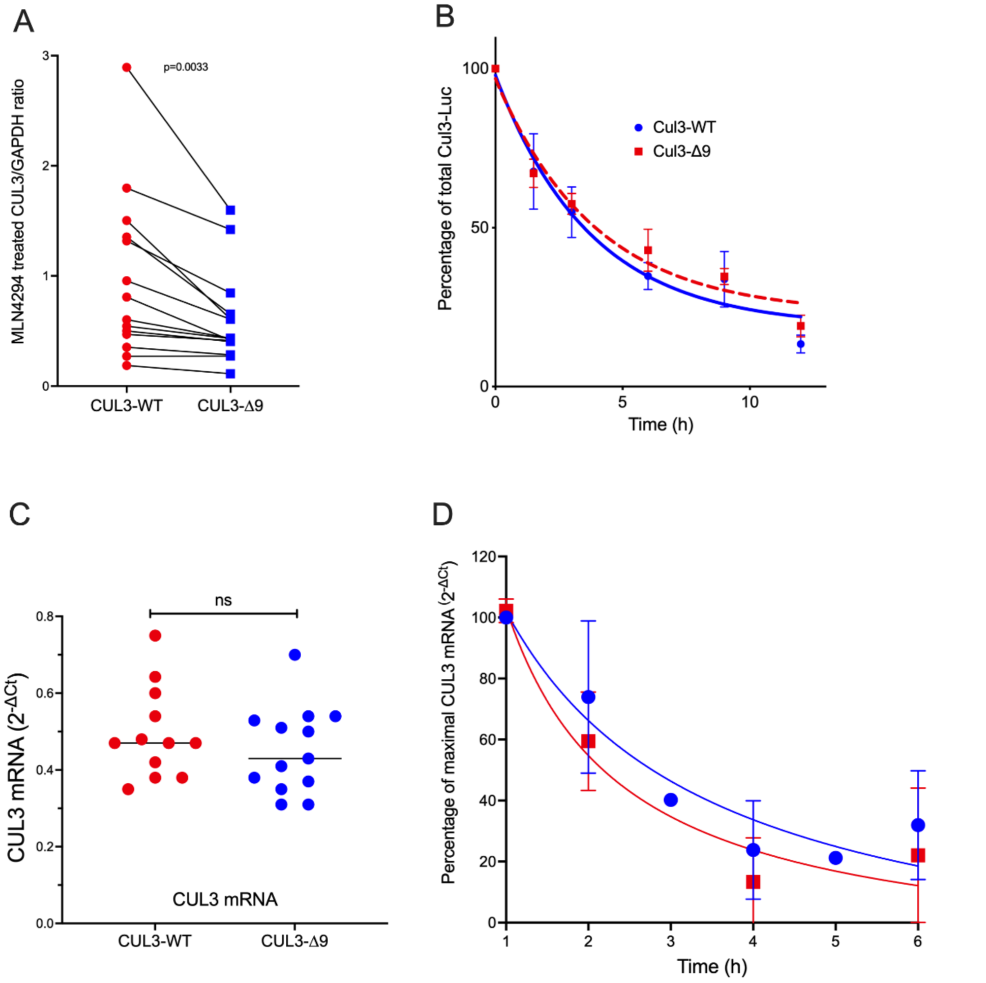

Figure S1. Expression of CUL3-WT and CUL3-Δ9 proteins and mRNA in stable Flp-In™ T-REx™ 293 cells.

A. The amount of CUL3 protein expressed in each cell line was measured by Western blot (n=14) on the un-neddylated (i.e. MLN4294 treated) forms of the WT and mutant proteins loaded on the same gel and related to the GAPDH amount (CUL3/GAPDH ratio). Results were compared by a paired Student t test. B. For CUL3 protein degradation, CUL3-WT and CUL3-Δ9 were tagged with luciferase and expressed in HEK293 cells. Twenty-four hours post-transfection, cells were treated with cycloheximide and collected at different time points. Luminescence of CUL3 was measured using a Mithras LB940 plate reader. Results were expressed as the percentage of the initial luminescent signal as a function of time (h). Similar decreasing curves were observed for CUL3-WT and CUL3-Δ9 with a half-life of 2.45±0.63h and 2.67±0.59h respectively (not significant p=0.854 (t.test)).

C. CUL3 mRNA content in the 2 cell lines was measured by RT-qPCR. RNA was extracted from cell pellets (0.5x10^7 cells) by RNeasy®Mini Kit QIAGEN (cat. nos. 74104) and was eluted by 50 µl RNase-free water. RNA (200 ng) was retrotranscripted by iScript™ cDNA Synthesis Kit (BIO-RAD) and qPCR was conducted using iQ™ SYBR® Green Supermix BIO-RAD on CFX96 thermocycler BIO-RAD (95°C 3min and 40 cycles of 95°C 15 sec, 60°C 30 sec). CUL3 mRNA was quantified by qPCR with a forward primer in the protein C tag (5′gcaccatcaccatcatcacg3′) and a reverse primer in the 5′ part of the CUL3 coding sequence (5′cagaaggtcccaaatgctgt3′). Three housekeeping genes were measured in parallel (HRPT (5′ctcaactttaactggaaagaatgtc 3′ and 5′ tccttttcaccagcaagct 3′), 18S (5′ ccctgccctttgtacacacc 3′ and 5′ cgatccgagggcctcacta 3′) and GAPDH (5′ tgcaccaccaactgcttagc 3′ and 5′ ggcatggactgtggtcatgag). CT was obtained by CFX Software and analysis by dCT (Cul3 CT –housekeeping CT geometric mean).
D. The degradation of CUL3-WT and CUL3-Δ9 mRNA was analyzed with the same RT-qPCR technic on cell pellets submitted to 10mg/mL of actinomycin D (Sigma) for 1, 2, 4 and 6 hours.

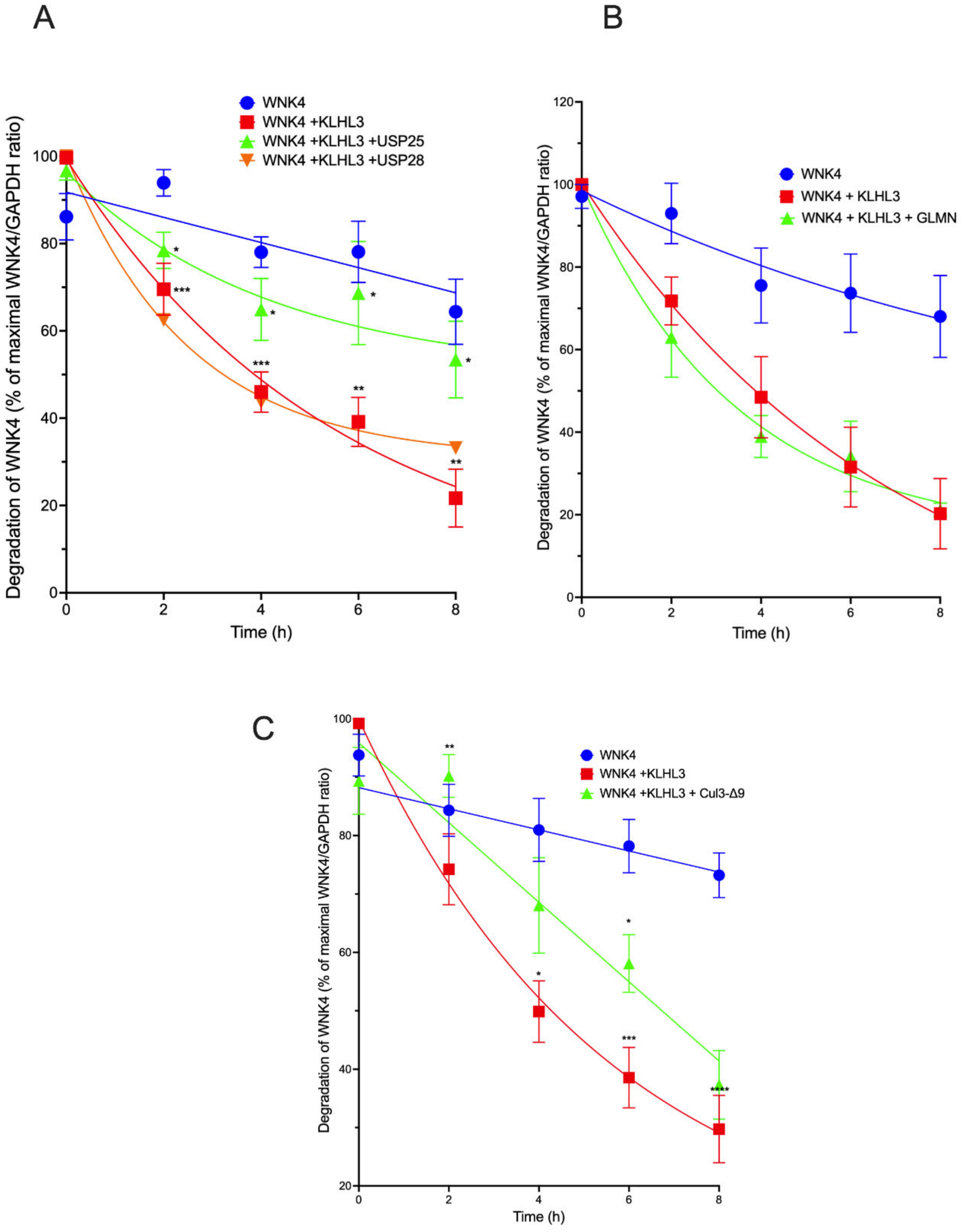

Figure S2. ©Flag-WNK4 was transiently transfected in cells with or without Flag-KLHL3, GFP-USP25 or HA-CUL3-Δ9. Six hours after transfection, cells were trypsinized and seeded in 6-well

plates. Two days post-tranfection, cells were treated with cycloheximide and collected at 2, 4, 6 and 8 hours post-treatment. Expression of Flag-WNK4 was analyzed by Western blot.

A Flp-In™ T-REx™ 293 cells expressing inducible PrC-KLHL3 protein were transfected with Flag-WNK4 ± GFP-USP25 or GFP-USP28. KLHL3 expression was induced or not by tetracycline 12h before cycloheximide treatment.

B Flp-In™ T-REx™ 293 cells expressing inducible PrC-GLMN protein were transfected with Flag-WNK4 ± Flag-KLHL3. GLMN expression was induced or not by tetracycline 12h before cycloheximide treatment.

C Flp-In™ T-REx™ 293 cells expressing inducible PrC-KLHL3 protein were transfected with Flag-WNK4 ± HA-CUL3-Δ9. KLHL3 expression was induced or not by tetracycline 12h before cycloheximide treatment.

WNK4 + KLHL3 was compared to WNK4, WNK4+KLHL3+USP25 and WNK4+KLHL3+CUL3-Δ9. * = $p < 0.05$, **= $p > 0.01$, ***= $p < 0.001$ and *** *=$p < 0.0001$ (unpaired t tests).
